# The role of testosterone in colorectal carcinoma: pathomechanisms and open questions

**DOI:** 10.1186/s13167-016-0071-5

**Published:** 2016-11-10

**Authors:** Mohsin H. K. Roshan, Amos Tambo, Nikolai P. Pace

**Affiliations:** Department of Anatomy, Biomedical Sciences Building, University of Malta, Msida, MSD2080 Malta

**Keywords:** Testosterone, Androgens, Colorectal cancer, Sertoli cells, Estrogen receptors, Biomarker, Risk prediction, Androgen receptor repeats

## Abstract

Colorectal cancer (CRC) is the fourth commonest type of malignancy after breast, lung and prostate in the USA and accounts for approximately 49,190 deaths annually in USA alone. The 5-year survival rate of CRC has increased over the past decades, in part, due to greater awareness and the widespread implementation of national screening programmes. Recently, a number of studies reported that males have a higher risk of developing CRC due to the action of testosterone.

Testosterone is an androgen that is responsible for the development of male secondary sex characteristics and for spermatogenesis. Studies on rats with mutated *Apc* tumour-suppressor gene subjected to either ovariectomy or orchidectomy exhibit different risks of CRC. Female rats subjected to ovariectomy are at higher risk of CRC, whereas orchidectomised male rats exhibit a lower risk of developing CRC. Sex hormones, in particular estrogen and testosterone, play a significant role in the development of CRC since the anti-neoplastic effect of estrogen lost during ovariectomy increases the risk of females developing CRC. Male mice exposed to testosterone after orchidectomy were also at greater risk than those who were orchidectomised but administered placebo only. Moreover, the recently established role of membrane androgen receptors in regression of CRC via non-genomic androgen-dependent action sets these receptors apart from intracellular androgen receptors (iARs) which themselves promote CRC development. In addition, testosterone-albumin conjugates are selective to membrane androgen receptors (mARs) and lead to apoptosis via caspase-3 activation. Akt kinases promote invasion of colon cancer cells when phosphorylated. These kinases are dephosphorylated upon activation of mARs, thereby reducing colon cancer cell motility and invasiveness.

Testosterone similarly plays important roles in human CRC. Long cytosine-adenine-guanine (CAG) repeats in the gene for the androgen receptors have been associated with a poor 5-year survival compared to shorter CAG repeats. Very recently, the measurement of serum unbound testosterone has been suggested as a novel biomarker along with carcinoembryonic antigen in CRC. In conclusion, testosterone may promote the development of CRC via a number of pathways, which may place males at greater risk. Testosterone holds promise as a potential biomarker in CRC risk prediction; however, further studies are required to better define its role in colorectal neoplasia.

## Background

Colorectal cancer (CRC) accounts for an estimated 49,190 deaths annually in the USA and is the fourth commonest type of malignancy after breast, lung and prostate. According to the National Cancer Institute, the prevalence of CRC is highest in the 65–74 age band, with a median age at diagnosis of 68 years, irrespective of race and gender. The 5-year relative survival rate of CRC has increased from 48.6 % in 1975 to 67.2 % in 2008, possibly attributed to increased awareness and the widespread implementation of CRC screening programmes [1].

Anatomically, the colorectum refers to the area of the distal gastrointestinal tract between the caecum and the rectum. It is anatomically divided into right and left sides. Clinically, it is divided into proximal to distal, this reflects the different embryological origins. The proximal colon is derived embryologically from the midgut and includes the caecum, ascending colon and proximal two thirds of the transverse colon. The distal colon is a derivative of the hindgut and includes the distal third of transverse colon, descending colon, sigmoid colon and rectum. Of CRC cases, 90–95 % are sporadic, and their development is described using Vogelstein model of carcinogenesis [2]. This model suggests that there is a transition from normal mucosa to adenoma initially, followed by the subsequent development of invasive carcinoma and metastasis due to sequential accumulation of deleterious mutation in genes which include *APC* (adenomatous polyposis coli), *K-ras* and *p53.* Gene inactivation by abnormal methylation patterns is also implicated in the development of CRC [3]. Approximately 10 % of colorectal cancers arise due germline-inherited mutations. Examples include familial adenomatous polyposis (FAP) and hereditary non-polyposis colorectal cancer (HNPCC). HNPCC arises as a result of inherited mutations in DNA mismatch repair genes such as MutL homolog-1 (*hMLH1*). Defects in the mismatch repair process leads to repetitive DNA sequences known as microsatellite instability (MSI) scattered throughout the genome [4]. The precise aetiology of CRC remains unknown, but certain risk factors may predispose an individual to CRC including high consumption of saturated fats and high dietary folic acid [5]. However, a recent meta-analysis concluded that no relationship exists between high folic acid intake and development of CRC [6].

The clinical presentation of CRC varies and depends on both the location of the primary tumour and the stage of the disease but may range from altered bowel habits to melena, with other systemic manifestations including anorexia and weight loss. Evidence shows that right-sided CRC is often associated with a poor prognosis compared to left-sided CRC, possibly due to the late clinical presentation seen commonly in right-sided CRC [7]. This review focuses on the relationship between testosterone and CRC in males and postmenopausal females and their risk of developing CRC. It also seeks to integrate and analyse current knowledge on testosterone in colorectal neoplasia from the predictive, preventive and personalised medicine context.

## Testosterone and its functions

### Classical functions of testosterone

Testosterone is a physiologically important androgen in both males and females but plays a significant role in the development of reproductive organs in the male. It is derived from cholesterol via the classical pathway, involving production of pregnenolone, 17α-hydroxyprognenolone and dehydroepiandrosterone. Testosterone acts via the androgen receptor NR3C4 (nuclear receptor subfamily 3, group C, member 4). This is a DNA-binding transcription factor which regulates gene expression and protein synthesis and plays a central role in increasing intracellular protein levels. As a result of androgen signalling, tissues proliferate and both basal metabolic demand and energy consumption increase.

Testosterone is metabolised to estradiol by the action of aromatase, particularly in females and obese individuals. Testosterone in males is produced mainly by the smooth endoplasmic reticulum of Leydig cells in the testes. In females, testosterone is mainly produced by the ovaries, suprarenal glands and adipose tissue. In the latter, extraglandular aromatisation of testosterone to estradiol and androstenedione to estrone occurs [8]. The anterior pituitary-derived hormones luteinising hormone (LH) and follicle-stimulating hormones (FSHs) are involved in the complex regulation of testosterone production and release. Studies have also reported that low molecular weight signalling molecules such as nitric oxide (NO) can affect the production of steroids by Leydig cells. Low concentrations of NO stimulate steroid production while high concentrations inhibit their production [9]. In addition, the inhibitory effect exerted by NO maybe potentiated via inhibition of the heme-containing steroidogenic enzyme CYP17 [P45oc17]. At higher concentrations of NO, Leydig cell function was inhibited through CYP11A1 [10]. Moreover, plasma levels of testosterone are affected by circadian patterns, as levels peak during sleep. Studies have suggested that hypotestosteronism may affect the quality of sleep and that large doses of exogenous testosterone may contribute to abnormal sleep durations [11]. Testosterone has also been shown to decrease neuron differentiation in rat embryos. This action is mediated by the androgen receptor (AR), which is expressed in large numbers by rat neuron cells; nonetheless, such findings have not yet been reported in humans [12]. Testosterone production can be influenced by both endogenous and environmental factors. For instance, drugs such as 5-fluorouracil have been reported to exert toxic effects on the male reproductive system in rats, resulting in a decreased serum levels of testosterone [13].

Aside from its classical functions as an androgen, the endogenous production of testosterone is also important for a healthy cardiovascular system. Numerous studies have shown that males with low levels of testosterone were prone to cardiovascular diseases [14, 15]. Furthermore, low levels of endogenous testosterone are directly linked to male infertility, erectile dysfunction and oligospermia. Conversely, the European Medicine Agency (EMA) Pharmacovigilance Risk Assessment Committee have expressed concern at the potential cardiovascular risks associated with testosterone replacement therapy [16]. Amos-Landgraf et al. have also demonstrated that testosterone may promote early colonic adenomagenesis in rats via as yet unknown mechanisms [17]. The link between testosterone and CRC development places males and postmenopausal women at higher risk of developing CRC and is further discussed in this review.

### Non-classical functions of testosterone in Sertoli cells

Testosterone plays an essential role in maintaining spermatogenesis and male fertility. In its absence, spermatogenesis is halted at meiosis. Moreover, mature sperm cells require testosterone in order for them to be released from the Sertoli cells. The classical actions of testosterone are widely described in the literature where testosterone activates gene transcription via translocation of androgen receptors and binding to specific DNA regulatory element.

AR knock-out mice exhibit infertility since the integrity of the blood-testis barrier (BTB) is compromised, exposing post-meiotic germ cells to autoimmune attacks and cytotoxic factors [18]. Moreover, there is also premature detachment of spermatids from the Sertoli cells in AR-deficient mice. Studies using Sertoli cells have confirmed that testosterone activates a number of kinase signalling pathways, which then alters the expression of genes that are not dependent on androgen response elements (AREs) or AR-promoter interactions [19]. These non-classical signalling pathways lead to the depolarisation of Sertoli cells by mediating Ca^2+^ influx via L-type Ca^2+^ channels. This results in altered transcriptional activity, an increase in secretions required by germ cells and stabilisation of cytoskeletal attachment between Sertoli cells and the developing germ cells [20]. In addition, stimulation of Sertoli cells by testosterone results in activation of a number of kinases and the consequent phosphorylation of CREB transcription factor, inducing CREB-mediated transcription and ERK kinase activity, thereby maintaining spermatogenesis. FSH blocks testosterone-mediated phosphorylation of ERK via cAMP and protein kinase A (PKA). Nonetheless, FSH does not inhibit CREB; instead, it induces CREB phosphorylation by increasing cAMP and PKA in order to maintain Sertoli cell support and survival of spermatocytes [21, 22]. These cells not only supply nutrients to developing germ cells, they are also responsible for maintaining constant supply of growth factors [23]. The BTB is maintained by testosterone via unknown mechanisms; this effectively creates a barrier between the adjacent Sertoli cells separating post-meiotic germ cells from external environment, therefore supporting their development [19].

## Role of testosterone in colorectal carcinoma

### Testosterone and colorectal cancer

The reported incidence of CRC is slightly higher in males than in females in the USA, and according to Arnold et al., the global incidence of CRC is expected to increase by up to 60 % by 2030 [24, 25]. Recent studies have suggested that testosterone may promote colorectal adenoma formation via unknown mechanisms, which may account for the observed higher male susceptibility to CRC. Mouse models are widely used to study the function of oncogenes and to investigate the genetic susceptibility to cancer. A mouse model of multiple intestinal neoplasia [Min] is generated by introducing specific mutations in the murine homolog of adenomatous polyposis coli (*Apc*) gene using conditional gene-targeting systems [26–28]. These heterozygous mice carry a truncated *Apc* gatekeeper tumour-suppressor gene at exon 15 which is exclusively mutated in human forms of colorectal adenomas and CRCs [17]. Nonetheless, most of these mice develop tumour primarily in the small intestine but not in the colon. Hence, a second rat model with a different nonsense mutation in the rat *Apc* gene, polyposis in the rat colon (Apc ^Pirc/+^), was generated which develops adenoma in the colon similar to that seen in humans. In addition, Pirc male rats are more susceptible to chemical induction of colorectal tumours with the carcinogen dimethylhydrazine [29]. In an elegant study by Amos-Landgraf et al., Apc^Picr/+^ rats were randomised within litters and were subjected to ovariectomy (OVX), orchidectomy (ORX) or sham control operation. They were either administered female hormone replacement [which included medroxyprogesterone acetate (MPA), estradiol (E2) and a combination of MPA and E2] or male hormone replacement therapy (which included dihydrotestosterone) or placebo via slow release pellets which were implanted subcutaneously in the nape of the neck. In addition, male mice who underwent ORX were administered testosterone enanthate in order to supplement the male hormone. Females that underwent OVX received either female hormone replacement therapy (HRT) or placebo whereas males received male HRT or placebo after ORX. As a result, orchidectomised males administered with placebo had a lower tumour load, which was comparable to that observed in females. In addition, the tumour load was lower in orchidectomised males in comparison to orchidectomised males administered with male HRT. This study demonstrated that male sex hormones, in particular testosterone, may play a role in inducing colonic adenomas. Furthermore, in order to assess the tumour-promoting role of male sex hormones, another set of mice were injected with the carcinogen azoxymethane (AOM) for 6 weeks. After this period, the investigators showed that male mice were more susceptible to developing adenomas compared to females injected with AOM [17]. The same male mice were then either orchidectomised or underwent sham operation and were administered further AOM injection. Orchidectomised mice developed substantially lower number of adenomas compared to those that underwent sham operation. A further experiment was carried out on orchidectomised male mice with a set receiving placebo while other receiving intramuscular injections of testosterone enanthate. The results confirmed the findings that testosterone somehow increases the susceptibility of males developing adenomatous lesions compared to those receiving only placebo [17].

In contrast to the findings reported in males, female mice that underwent ovariectomy developed more adenomatous lesions compared to those who underwent sham operations. This may be attributed by the loss of protective effect by estrogen in ovariectomised females in suppressing adenomas. The findings reported by Amos-Landgraf et al. support the notion that female sex hormones may have protective effects against CRC while male sex hormones may actively promote CRC development [17, 30]. The anti-neoplastic properties of estrogen have been reported in a number of studies, where the number of colonic adenomatous lesions was reduced in ovariectomised female mice administered 17β-estradiol [31]. Moreover, genetic inactivation of one or both estrogen receptors (Er) may contribute to the development of CRC in females [17]. Nonetheless, contradictory results have been reported suggesting the strain of mice used and their underlying genetic susceptibility to carcinogens may attribute to the differences in results [32, 33].

Testosterone has been shown to exert pro-apoptotic effects in both prostate and colon cancer cells via a membrane-initiated mechanism [34]. Dehydroepiandrosterone (DHEA) can control cell fate, activating nerve growth factor (NGF) receptors, mainly tropomyosin-related kinase (Trk)A and p75 neurotrophin receptor in primary neurons and in PC12 (phaeochromocytoma) tumour cells. NGF promotes cancer cell proliferation and apoptosis. Recently, it was shown that DHEA and NGF strongly blunt serum deprivation-induced apoptosis, whereas testosterone induces apoptosis of cancer cells, contrary to findings reported by Amos-Landgraf et al. [34]. Moreover, the anti-apoptotic effects of both DHEA and NGF were completely reversed by testosterone. It appears testosterone is able to block DHEA and NGF receptors, thereby antagonising their actions [34].

### Androgen receptors in CRC signalling

Colon tissues express functional nuclear steroid receptors; specifically, androgen receptors (ARs), estrogen receptor-α (ERα) and estrogen receptor-β (ERβ) have been reported expressed in CRC [35]. ARs are the binding site for dihydrotestosterone (DHT), and two isoforms of the AR, androgen receptor A (AR-A) and androgen receptor B (AR-B) [110 kDa], were reported by Catalano et al. [36]. Both of these were detected in healthy colonic mucosa whereas only AR-A (an 87-kDa isoform) was detected in neoplastic colonic mucosa. Neoplastic colonic mucosa is characterised by loss of expression of the AR-B isoform, whereas AR-A expression is maintained [36]. Low levels of AR-A are normally detected in the fetal colon, whereas AR-B is expressed in both the fetal and adult colons. However, the expression of AR-A in CRC may be indicative of loss of cell differentiation [36]. Recently, it was reported that membrane androgen receptors (mARs) expressed in colonic tumours may trigger apoptosis in neoplastic cells. mARs are cell-surface G-protein coupled receptors (GPCR) which signal via modulation of intracellular calcium or inositol 1,4-5-triphosphate second messenger pathways. The non-genomic androgen-dependent action of these mARs sets them apart from the intracellular androgen receptors (iARs) which mediate signalling through dimerisation, nuclear translocation and activation of androgen-specific target genes [37]. Membrane androgen receptors may induce tumour regression, in contrast to iARs according to Gu et al. [38]. Colon cancer tissues isolated from mice xenograft tumours and two colon cancer cell lines (Caco2 and HCT116 cells) were used in this study by Gu et al. Significant mAR expression were reported in xenograft tumour tissues and cell lines compared to healthy or non-transformed cells. Furthermore, when human epithelial colorectal adenocarcinoma cells (Caco2 cells) were incubated with either radiolabeled testosterone, varying concentrations of DHT or estradiol, the radiolabeled testosterone was displaced by DHT. These radioligand binding studies suggest high affinity and selectivity of mARs towards specific androgens [39]. Furthermore, fluorescence staining of Caco2 and HCT116 cells treated with testosterone-3-[O-carboxymethyl] oxime human serum albumin (testosterone-HSA) for 24 h revealed evidence of apoptosis via caspase-3 activation. These reported effects were solely attributed to the activation of mAR expressed in colon cancer cells. As mARs are not expressed in non-neoplastic colon cells, these do not respond to testosterone treatment compared to colon cancer cells. No iARs were detected in membrane preparations of colon cancer cells nor were involved in any part of the study by Gu et al. The study concluded that colonic tumour cells may over-express pro-apoptotic pathways in an mAR-dependent fashion leading to tumour regression and that mARs may present novel pathways for pharmacologic therapy in colorectal neoplasms [39].

Aside from mAR expression, protein kinase B (Akt) is a serine/threonine-specific protein kinase which plays a major role in the invasiveness of colon cancer cells in response to a variety of stimuli ranging from heregulin, PAK1 and Sprouty-2 [40–42]. Phosphorylated Akt (p-Akt) is upregulated in colonic tumour cells but not in non-transformed cells, and is dephosphorylated following long-term mAR activation with testosterone-albumin conjugate (TAC) treatment both in vitro and in vivo [38]. As a result, the upstream regulator of Akt, in particular PI-3K, is dephosphorylated upon long-term TAC treatment, thereby reducing cell motility in colon cancer and its invasiveness. In addition, control experiments using colon tumour cells treated with either testosterone or HSA alone show that this does not downregulate p-Akt when compared to testosterone-HSA treatment. This apparent contraindication is in line with the dual role of testosterone in simultaneous binding of mARs [thereby inducing p-Akt downregulation] and iARs [thereby inducing p-Akt upregulation] within the same cells. Vinculin, a cytoskeletal protein which links integrin adhesion molecules to actin, was reported as the main target of mAR activation that may regulate cell motility since inhibition of vinculin via phosphorylation promotes colonic cancer cell migration [38].

### Length of cytosine-adenine-guanine repeats in androgen receptor gene and CRC

CRC is known to develop through a number of genetic mechanisms, which include microsatellite instability and suppression of *APC* gene activity. The growth of cells in colonic mucosa is in part regulated by androgens including testosterone. The gene encoding the AR is located on chromosome Xq11-12 and contains varying number of CAG repeats (8-35 CAG repeats) in normal populations. These CAG can alter the function of the AR gene. A case-control study investigating the effect of this polymorphism on CRC risk was carried out on 550 CRC cases and 540 healthy controls, who were analysed for presence of CAG sequences in the AR gene using polymerase chain reaction (PCR) [43]. The study showed that the CRC group had a longer CAG repeat length compared to the control group and that this repeat confers an increased risk of developing CRC in both males and females, whereas a shorter CAG repeat size may protect individuals against CRC. In addition, differences in CAG repeat may determine the survival rate in CRC. Cases with long CAG repeat sequences had a poor 5-year survival compared to those with shorter CAG repeats. In addition, the absence of AR expression in long CAG repeat sequences was associated with tumour size larger than 5 cm in diameter, which were moderate to poorly differentiated (at T3–T4 and N1–2 stages) compared to those with normal expression of AR and short CAG repeat sequences. Aside from the link to tumour stage, these patients were also reported to have a poor survival rate even after postoperative adjuvant chemotherapy as they demonstrated greater risk of recurrence or metastasis [43]. On the contrary, a different study has reported that there is no association between the length of CAG repeats in the AR and colorectal cancer. Similarly, the CAG repeat in the AR showed no association with the overall survival rate. Instead, an association between the estrogen receptor 2 (ESR2) CAG repeat polymorphism was reported to be associated with increased risk of CRC among women but not men [44].

A further study investigating the role of the ESR2 CAG repeat reported a sixfold increased risk of CRC in women harbouring two ESR2 short alleles [less than 22 repeats] compared to women harbouring two long alleles (more than 22 repeats) [45]. Aside from CAG repeats, co-activator-associated arginine methyltransferase 1 (CARM1), a protein with arginine-specific histone methyltransferase activity, has been reported to promote nuclear receptor activity and act as a molecular switch that controls gene-specific transcription factors including p53, NF-ĸB, LEF1/TCF4 and E2Fs. It primarily binds to the histone and p160 co-activators in order to activate nuclear receptors which includes ARs [46, 47]. In doing so, CARM1 plays a significant role in cell proliferation and survival [48, 49]. Overexpression of CARM1 in CRC was reported in CRC specimens but was weakly expressed in normal mucosal cells. Furthermore, CARM1 is believed to modulate transcription of p53, a tumour-suppressor gene, as well as of NF-ĸB. CARM1 inhibits the p53 response and promoted the NF-ĸB response in Caco2 cells via unknown mechanisms [50]. More molecular and genetic profiling of CRC is required to identify the precise role of these proteins.

### Sex hormones and gender differences in CRC

Age is one of the strongest risk factors for the development of CRC, with 90 % of cases reported in individuals over the age of 50. However, the incidence of CRC is lower in women across all age groups [51]. Female sex hormones are known to have a protective role against the development of a number of diseases, including cardiovascular disease (CVD) as well as CRC according to the Woman’s Health Initiative (WHI) study [17]. A recent randomised controlled trial in women on hormone replacement therapy over a period of 5 years showed a substantial reduction in CRC in women on equine estrogen (E2) and medroxyprogesterone acetate (MPA) compared to placebo [52]. Following this clinical trial, a subsequent investigation was conducted in females who had previously undergone hysterectomy and were administered E2 only. Surprisingly, the second trial showed that women administered E2 were not protected against the development of CRC. This suggests that MPA, in particular progestin, may play a protective role in females [53]. Nonetheless, the precise mechanism is unknown and requires further investigations. The hormonal differences in different genders do not end there since a number of studies have shown that female patients respond better than male patients to adjuvant chemotherapy in a number of cancers. These observations suggest that gender and hormones play a central role in the development of CRC and its prognosis.

Female sex hormones confer a protective effect against CRC development. Estrogen is a member of the steroid hormone family and is usually associated with female reproductive function. 17β-estradiol (E2) is the most potent estrogen in humans when compared to estrone (E1) and estriol (E3). Estrogen functions are mediated by two nuclear receptor subtypes, estrogen receptor α (ERα) and estrogen receptor β (ERβ), which then bind to genomic regulatory regions known as estrogen response element (ESR) either as a homodimer or heterodimer. This then results in transcription and activation of a number of genes (VEGF, TGF-β, cadherins, laminins) which are involved in angiogenesis, cell adhesion and apoptosis. Non-genomic effects of estrogen are independent of DNA interactions; instead, it modulates cellular processes via activation of diverse intracellular pathways including protein kinase C (PKC), intracellular Ca^2+^, cytosolic cAMP, NO and MAPK [51]. ERβ is considered to confer the protective effects of estrogen against CRC as they are primarily found in normal colonic epithelium and their expression declines in CRC cells [54, 55]. Moreover, colorectal cells and even cancer epithelial cells do not co-express ERα and ERβ. Studies have shown ERβ expression results in inhibition of proliferation and G_1_ phase cell-cycle arrest [56]. Moreover, ERβ inhibits cMyc and tumour growth in a mouse model, suggesting ERβ may have anti-proliferative properties which are not seen with ERα [57]. Nonetheless, the exact role of estrogen and ERs in CRC is still unknown and some studies have suggested that menopausal women may have a higher risk of CRC due to lower levels of estrogen, as well as due to loss of cell cycle regulatory effects of ERβ. This is evidenced by studies which show that the use of oral contraceptives and HRT treatment in postmenopausal female translates to lower reported incidences in CRC [51]. Trials carried out by the WHI study in females administered with estrogen in combination with progestin reported a 40 % lower risk of CRC compared to those on placebo [52].

On the other hand, males having lower androgenicity as a result of longer CAG repeats in the AR gene or on treatment with androgen deprivation therapy were associated with a high reported incidence of CRC. Thus, the protective effects of estrogen and progesterone in women and testosterone in men against CRC is clinically important. Studies have shown that males tend to develop colonic lesions at an earlier age compared to females who often develop neoplastic changes at much later stages in life [58]. Moreover, males on androgen deprivation therapy with gonadotropin-releasing hormone (GnRH) agonists for prostate cancer treatment have an increased risk of CRC. Furthermore, males who underwent orchiectomies were reported to have higher incidences of CRC followed by those on GnRH agonist therapy especially if they are on androgen deprivation treatment for a long period of time [59]. The results from these studies suggest that testosterone confers some degree of protection against CRC and contradicts other investigations which suggest that testosterone may promote CRC [17, 34, 60]. In addition, high levels of testosterone have been associated with an increased risk of early death after cancer diagnosis in both males and females, but such findings have not been reported in CRC [61]. Males with higher levels of testosterone and sex hormone-binding globulin (SHBG) but low levels of estradiol were shown to have a lower risk of developing CRC. This suggests that males with lower androgenicity due to reduce AR activity or low circulating androgens were at greater risk of CRC; the risk is greater in males undergoing androgen deprivation therapy [59]. Moreover, obese males, particularly those with a higher ratio of estradiol to testosterone, have an increased activity of aromatase that therefore may predispose these individuals to CRC. Estradiol relays negative feedback response which inhibits secretion of LH and subsequently testosterone release [62]. Therefore, contrary to females, it can be concluded that estradiol confers no protection against CRC in males.

Premenopausal females normally have a higher ratio of estradiol to testosterone levels as a result of higher aromatase activity leading to increased production of estradiol. This therefore leads to a lower risk of developing CRC in females when compared to males [63]. Aromatase activity also offers protective effects in postmenopausal females by increasing the ratio of estradiol to testosterone, but levels of estradiol are not as high as those seen in premenopausal females. Nevertheless, these aromatase-dependent beneficial effects on CRC risk are only observed in non-obese females but not in obese females [63].

### Testosterone as a tumour marker in CRC

Hypotestosteronemia—defined as low levels of testosterone (11 nmol/L or 320 ng/dL)—has been reported to be a contributing factor in the development of CRC [64]. This makes quantification of serum testosterone an attractive potential biomarker for prediction of CRC risk. A study by Holland et al. evaluated concentration of testosterone in patients with histologically confirmed CRC. They quantified serum testosterone, estradiol and carcinoembryogenic antigen (CEA) in CRC patients before and after undergoing surgical resection. The results were then compared with the control group who were carrying the benign gastrointestinal pathologies. Their results showed that unbound serum testosterone was lower in those with colon or rectal neoplasia and 56.9 % of CRC cases had elevated CEA levels [65]. The combined use of both of these biomarkers increased the sensitivity of tumour screening compared to the use of CEA in isolation. The authors concluded that quantification of testosterone in combination with CEA may enhance tumour screening and suggested that it could be used for diagnosis and follow-up of CRC, especially in CRC patients with normal CEA [65]. Hypogonadal males with high levels of prolactin might be at a greater risk of CRC since prolactin is reported to exert mitogenic effects which may confer to tumorogenecity. Hence, prolactin in conjunction with testosterone may be a useful tumour marker in CRC [66]. Serum testosterone is already in clinical use as a biomarker for prostate cancer and has been proposed as a tool for measuring prognosis of breast cancer in postmenopausal women [67]. Nonetheless, data of its use in CRC is limited and emerging evidences on the role which testosterone plays in CRC warrants further long-term double-blinded clinical trials.

### Testosterone and CRC—from biology to predictive and personalised medicine

The experimental, epidemiological and clinical studies outlined earlier in this review provide compelling evidence that androgens are involved in the process of CRC development. Although CRC is not a hormone-dependent cancer in the classical sense, the role of sex hormones in its pathogenesis has potential translational applications in pre-symptomatic diagnosis, risk stratification and treatment. The implications of this body of knowledge are extensive, particularly in the light of shifting trends towards predictive, preventive and personalised medicine (PPPM) in oncology. The ultimate aim of PPPM in cancer is to improve patient outcome through the implementation of rapid, sensitive and specific new methods for tumour detection [68]. This requires the development and validation of predictive and prognostic biomarkers to guide diagnosis and therapy. Current screening and diagnostic protocols for CRC are based on colonoscopy, biopsy and histologic examination of tissue samples. Furthermore, CRC is nearly asymptomatic in its earliest stages. This combined scenario of asymptomatic early disease and invasive diagnostic procedures reinforces the need for the development of novel biomarkers to facilitate screening and the identification of individuals at risk. A detailed overview of existing and emerging molecular biomarkers of CRC is available in the literature and is beyond the scope of this review [69].

The quantification of serum testosterone as an adjunct to existing biomarkers for CRC is an attractive prospect. The availability of direct automated chemiluminescent assays for multipurpose immunoassay platforms facilitates measurement of testosterone in routine clinical chemistry laboratories, rendering the process rapid and convenient. However, some authorities have been highly critical of the accuracy and precision of routine immunoassays when compared to gold-standard liquid chromatography/tandem mass spectrometry (LC/MSMS) platforms [70]. Wang et al. showed that routine immunoassays should not be used to measure testosterone in the normal female or paediatric ranges [71]. Similarly, Moal et al. showed that no immunoassay is reliable when low testosterone concentrations (≤3.47 nmol/L) are measured [72]. The controversial nature of this issue dictates the burgeoning need to better define the sensitivity of existing assays, inform clinicians of the implications of these limitations and implement the use of gold-standard platforms to improve sensitivity and accuracy.

The determination of androgen receptor CAG repeat length provides a second aspect of testosterone physiology that has potential translational application in the biomarker arena. Clearly, genotypes are attractive as disease biomarkers. They are independent of age and do not change, and technological advances allow for high-throughput multiplex genotyping assays that provide high fidelity and reproducibility. Furthermore, the potential integration of genotype-based data into a risk-prediction or prognosis-prediction algorithm for CRC lays the foundation for a personalised therapeutic approach addressed to a particular patient.

One of the key expert recommendations by the EPMA (European Association for Predictive, Preventive and Personalised Medicine) concerning cancer biomarkers provides a stark reminder of the complex mechanisms at play in cancer biology. The authors emphasise the point that cancer is a multifactorial, whole-body disease that leads to alterations in multiple cell pathways. Hence, a paradigm shift is required, moving from a traditional single-factor strategy to a multi-parameter approach [73]. When considering testosterone as a potential biomarker in CRC, this approach is highly relevant, as diagnostic or prognostic benefit might be obtained if testosterone levels or CAG repeat length are considered in combination with data from other biomarkers. This requires the development and validation of biomarker panels to provide meaningful data in risk prediction or stratification. However, one of the major challenges facing cancer biomarker development is the integration of large ‘omics’ datasets with clinical data and their subsequent interpretation and translational application. Clearly, the bench-to-bedside translation of multi-parameter biomarker panels remains a major hurdle and accounts for the significant discrepancy in numbers between the number of biomarkers in pre-clinical development and biomarkers in commercial use [74].

## Conclusions

CRC is one of the most common types of cancer with higher prevalence in those above the age of 50 [51]. The 5-year survival rate of CRC has improved over the years as a result of international awareness and better screening programmes. Testosterone as a male androgen responsible for the development of secondary sex characteristics as well as spermatogenesis has recently been reported to increase susceptibility of acquiring CRC in males. Such findings were supported by animal studies which concluded that orchidectomised mice have a lower risk of developing CRC compared to controls. On contrary, female mice who underwent ovarectomy were at a greater risk of developing CRC than their counterparts who had no ovarectomy [17]. These findings support the notion that sex hormones may confer protection against CRC or promote the development of CRC as the case with testosterone. Moreover, the recently discovered mARs are significant, since activation of these receptors occurs predominately in CRC but not in normal colonic mucosa. In addition, activation of these receptors by testosterone-HSA conjugates triggers apoptotic pathways that offer protection against CRC.

Tumour invasiveness is determined by Akt, a serine kinase which regulates the motility of colonic cancer cells and prevents their invasiveness [75]. The activities of these kinases are enhanced by testosterone-HSA, thereby inhibiting tumour invasion and distant spread. Aside from Akt kinases, the length of CAG repeats in the AR gene is equally important since long CAG repeats were associated with poor 5-year survival compared to short CAG repeats. Apart from the CAG tandem repeats, the CARM1 protein is important in cell proliferation and survival [50]. Overexpression of these proteins have been reported in CRC specimens, but their precise role and mechanisms are yet to be determined.

Other non-modifiable factors such as gender differences and different functions of sex hormones may explain why CRC cases are often reported in males. Females are often protected against CRC due to anti-cancer properties of estrogen and activity of aromatase which converts testosterone to estrogen. As a result, CRC cases are often presented much later in postmenopausal females compare to males [63]. The precise role of testosterone in CRC is not well understood, and a number of conflicting studies have been published on the relation between testosterone and CRC risk. Therefore, more long-term clinical trials, molecular and genetic studies are required in order to determine the precise role of testosterone in human colonic mucosa since most experimental data currently available is on animal models which may not full represent normal human pathophysiology. Recently, testosterone was proposed as a tumour biomarker for CRC in conjunction with other tumour markers currently available such as CEA. In doing so, it may increase the specificity of these tumour markers to allow better follow-up after surgical management of CRC. Despite the biological link between testosterone and CRC, the translational application of this knowledge into patient care requires further research. In particular, the use of testosterone as a cancer biomarker requires harmonisation of laboratory quantification methods to overcome issues of sensitivity and accuracy. Furthermore, diagnostic and prognostic models involving multiple biomarkers need to be developed and validated in different populations to determine whether testosterone has any clinical utility in CRC. The development of novel biomarkers for CRC should in part be based on sound biological knowledge about the in vivo function of the chosen molecules, and this requires better understanding of the cell pathways that are dysregulated in the development of colorectal neoplasia. CRC is a multifactorial disease that is affected by a multitude of risk factors, including the environmental exposure to chemicals, toxins and carcinogens, as well as dietary supplements. Therefore, a wider clinical picture should be obtained using data extrapolated from molecular studies and animal models in order to better determine the precise mechanism that drives CRCs.

## Abbreviations

AJCC: American Joint Committee on Cancer
Akt: Protein kinase B (PKB/Akt)
AOM: Azoxymethane
APC: Adenomatous polyposis coli
Apc ^Min/+^: Adenoma anaphase-promoting complex multiple intestinal neoplasia
AR-A/AR-B: Androgen receptor A and B
AREs: Androgen response elements
ARs: Androgen receptors
BTB: Blood-testis barrier
Caco2 cells: Colon cancer cell line 2
CAG: Cytosine-adenine-guanine
cAMP: Cyclic adenosine monophosphate
CARM1: Co-activator-associated arginine methyltransferase-1
CEA: Carcinoembryonic antigen
cMYc: Transcription factor regulatory gene
CRC: Colorectal cancer (carcinoma)
CREB: cAMP response element-binding protein
CVD: Cardiovascular disease
DHEA: Dehydroepiandrosterone
DHT: Dihydrotestosterone
E1: Estrone
E2: Estradiol
E3: Estriol
EPMA: European Association for Predictive, Preventive and Personalised Medicine
Er: Estrogen receptors
ERα: Estrogen receptor alpha
ERβ: Estrogen receptor beta
ESR2: Estrogen receptor 2
FAP: Familial adenomatous polyposis
FSH: Follicle-stimulating hormone
GnRH: Gonadotropin-releasing hormone
HCT116 cells: Human colon carcinoma cells 116
hMLH1: MutL homolog-1
HNPCC: Hereditary non-polyposis colorectal cancer
HRT: Hormone replacement therapy
HSA: Human serum albumin
iARs: Intracellular androgen receptors
LH: Luteinising hormone
MAPK: Mitogen-activated protein kinase
mARs: Membrane androgen receptors
MPA: Medroxyprogesterone acetate
MSI: Microsatellite instability
Na^+^/K^+^ ATPase: Sodium/potassium adenosine triphosphatase
NF-ĸB: Nuclear factor kappa-light-chain enhancer B cells
NGF: Nerve growth factor
NO: Nitric oxide
ORX: Orchidectomy
OVX: Ovariectomy
P53: Tumour protein 53
PC12: Pheochromocytoma
PKA: Protein kinase A
PKC: Protein kinase C
PPPM: Predictive, preventive and personalised medicine
SHBG: Sex hormone-binding globulin
Trk-A: Tropomyosin-related kinase A
WHI: Woman’s Health Initiative
